# Association between postnatal mental health-related hospitalisation and child development and education outcomes: a systematic review and meta-analysis

**DOI:** 10.3389/fgwh.2026.1721113

**Published:** 2026-05-28

**Authors:** Demeke Mesfin Belay, Abel Fekadu Dadi, Yohannes Tesfahun Kassie, Wubet Alebachew Bayih, Behailu Derseh, Binyam Minuye Birhane, Bernard Leckning, Steven Guthridge

**Affiliations:** 1 Menzies School of Health Research, Charles Darwin University, Darwin, NT, Australia; 2 College of Health Science, Debre Tabor University, Debre Tabor, Ethiopia; 3 Addis Continental Institute of Public Health, Addis Ababa, Ethiopia.; 4 Department of Epidemiology and Preventive Medicine, School of Public Health and Preventive Medicine, Faculty of Medicine, Nursing and Health Science, Monash University, Victoria, Melbourne, Australia; 5 Department of Public Health, Asrat Woldeyes Health Sciences Campus, Debre Berhan University, Debre Berhan, Ethiopia; 6 School of Public Health, University of Technology Sydney, Sydney, NSW, Australia; 7 Discipline of Psychiatry and Mental Health, School of Clinical Medicine, Faculty of Medicine and Health, University of New South Wales, NSW, Sydney, Australia

**Keywords:** development, education, hospitalisation, maternal mental health, postnatal

## Abstract

**Aim:**

This study consolidates existing knowledge regarding the association between postnatal mental health-related hospitalisation (MHrH) and childhood development and education outcomes.

**Methods:**

We searched MEDLINE, PsycINFO, CINAHL, Scopus, Embase, Google Scholar, and the reference list of accessed papers. A weighted random-effects meta-analysis was conducted. A trim and fill analysis was executed following the detection of publication bias. Quality assessment of included studies was performed using the Joanna Briggs Institute (JBI) tool.

**Results:**

Children whose mothers experienced postnatal MHrH had elevated odds of developmental vulnerability in any of five developmental domains (Pooled Odds Ratio (POR) = 1.53, 95% CI; 1.20–1.86), in the social domain (POR = 1.27, 95% CI; 1.18–1.35), emotional domain (POR = 1.34, 95% CI; 1.16–1.51), and physical domain (POR = 1.26, 95% CI; 1.14–1.38) compared to children whose mothers had no postnatal MHrH. Children whose mothers experienced postnatal MHrH had elevated odds of below-average academic performance on any of four measures (POR = 1.57, 95% CI; 1.07–2.07), spelling measure (POR = 1.48, 95% CI; 1.01–1.95), and writing measure (POR = 1.37, 95% CI; 1.04–1.70). A higher albeit statistically uncertain odds of developmental vulnerability in the cognitive domain was found to be associated with postnatal MHrH (POR = 2.41, 95% CI; 1.00–3.82). No statistically significant associations were observed between postnatal MHrH and vulnerability in the communication skill (POR = 1.21, 95%; CI 0.96–1.47), or with below-average academic performance in numeracy (POR = 2.02, 95%; CI 0.37–3.68) and reading (POR = 1.57, 95%; CI 0.59–2.55).

**Conclusion:**

This review found that children whose mothers experienced postnatal MHrH were at greater risk of vulnerability in at least one domain of development, as well as below-average performance in at least one area of academic outcomes. Whilst these results should be interpreted cautiously due to substantial heterogeneity among the included studies, there is sufficient evidence to support existing recommendations for early screening, diagnosis, and management of maternal mental health as part of routine postpartum care.

**Systematic Review Registration:**

https://www.crd.york.ac.uk/PROSPERO/view/CRD42023446155.

## Introduction

The World Health Organization (WHO) highlights the significance of mental health for overall well-being (1) and its role in achieving the Sustainable Development Goals (SDGs) (2). Maternal mental health-related conditions have been recognised as a significant public health concern affecting millions of women worldwide (3). Women at the time of pregnancy and following birth are at a higher risk due to unique stressors related to pregnancy and postpartum adjustments (4–6). Within the postnatal period, women experience various emotions, including joy and contentment, anxiety, frustration, sadness, and guilt, making them susceptible to multiple mental health-related conditions (7). While obstetric definitions typically extend the postpartum period to six weeks after delivery (8), it has been defined from four weeks to eighteen months for research purposes (9, 10); hence, our review considered primary studies that covered the postnatal period up to 18 months after delivery.

Globally, about 13% to 20% of women encounter postnatal mental health-related conditions (3). Pre-existing mental health-related conditions, conditions before and during pregnancy, are the most common risk factors for the emergence of mental health-related conditions within the postnatal period (11, 12). A frequently employed measure of mental health-related conditions at a population level is the rate of hospital admissions. Hospital admission rates for mental health-related conditions are significantly higher in the postnatal period compared to the antenatal period (11–14). Maternal mental health-related hospitalisation (MHrH) within the postnatal period primarily includes severe depression, schizophrenia, schizoaffective disorders, psychotic disorders, substance misuse, and bipolar disorders (15–17).

These conditions have been reported to have short and long-lasting consequences on the child’s development and education outcomes (18–22). The effects of MHrH on a child’s development outcomes (23–26) may be due to mothers’ reduced capacity to provide optimal care and emotional support for their children (27, 28). Observational studies have shown that children whose mothers have a MHrH within the postnatal period experience educational difficulties, including reduced classroom engagement, lower academic performance, increased school absenteeism, dropping out of school, and a greater likelihood of requiring special education (29–33). Maternal MHrH within the postnatal period also imposes a significant economic burden. For instance, in the United Kingdom, an estimated £8.1 billion has been spent annually on perinatal mental health, with a substantial portion going towards societal costs and child-related expenses (34).

Despite the high burden of maternal MHrH within the postnatal period and adverse impacts on child development and education outcomes, there is inconsistent and inconclusive reporting among studies (35, 36). Furthermore, based on searches of Cochrane, Epistomonikos, and PROSPERO, no systematic reviews or meta-analyses have sought to synthesise the evidence on the effects of postnatal MHrH on child development and education outcomes. To address this gap in the evidence and inform opportunities for prevention and intervention, we designed a systematic review and meta-analysis to assess the association between postnatal mental health-related hospitalisation and child development and education outcomes.

## Methodology

### Protocol registration and reporting

Consultation with experts in systematic review and meta-analysis methodology took place during the formulation of the protocol. The protocol was registered with the International Prospective Register of Systematic Reviews (PROSPERO) [CRD42023446155]. To uphold transparency and reproducibility, the systematic review and meta-analysis were reported according to the Preferred Reporting Items for Systematic Review and Meta-analysis (PRISMA) 2020 checklist (37) [Supplementary file (Supplementary Figure 1)].

### Data source and search strategy

Following advice from consultant librarians, specific search strategies were designed for each academic database (MEDLINE, PsycINFO, Embase, CINAHL, and Scopus) and Google Scholar from inception to August 2023. A final update search was performed on January 11, 2024, to capture any studies published/reported after our initial search. Additionally, a manual search examined cross-references and bibliographies from selected publications to uncover potentially relevant articles. A grey literature search was conducted using Google, following the PECO format with Medical Subject Headings (MeSH) terms, wildcards, and keywords related to postnatal MHrH, development, and education outcomes. We used Boolean operators, such as “AND” and “OR”, to combine search terms. A detailed summary of search terms and strategies is provided in the (Supplementary file [Supplementary file (Supplementary Table 1)].

### Eligibility criteria and outcome of interest

The review included observational studies (case-control, cohort, and cross-sectional) to address the following research question: Are children aged 0–18 years (population (P)) who were exposed to any maternal MHrH within the 18-month postnatal period (exposure (E)), diagnosed through clinical assessment, standardised tools, or medical records, and reported at least one developmental or educational domain (outcomes (O)) compared with children whose mothers have no record of MHrH within the postnatal period of up to 18 months (comparator (C))?.

We restricted the exposure to maternal mental health-related hospitalisation for the following reasons. First, this approach enhances diagnostic validity, as inpatient admissions typically involve comprehensive, multidisciplinary assessment and are systematically and reliably recorded. Second, inpatient admissions generally indicate more severe or recurrent mental illness that cannot be effectively managed outside hospital care. Such cases may have a strong association with adverse child outcomes. This review encompassed studies without restriction on language, geography, or year of publication. This review excluded reviews, commentaries, editorials, or conference abstracts. Exposure was defined as children whose mothers had at least one hospitalisation for any mental, behavioural, and neurodevelopmental disorders within the 18-month postnatal period. Developmental outcomes refer to measurable indicators of a child's development, often categorised as competencies in physical, cognitive, emotional, social, and communication domains. A child was classified as a case (i.e., developmentally vulnerable) if their score in any of the assessed developmental domains fell below the expected threshold. Educational outcomes relate to academic performance and are often defined across reading, writing, spelling, and numeracy measures. A child was classified as a case (below-average academic performance) if their achievement in any of the assessed areas fell below the expected academic standard on a given measure.

### Study selection

EndNote version 20 was used to manage published studies and eliminate duplicates (38). The study selection process involved screening titles and abstracts and full-text screening using the standardised Joanna Briggs Institute (JBI) tool (39). Two authors (DMB and YTK) independently screened the titles and abstracts to identify those meeting the inclusion criteria. Simultaneously, two other authors (BMB and BD) screened titles and abstracts that were deemed ineligible or excluded after the initial screening to identify additional eligible studies. One author (WAB) examined reference lists to identify potentially relevant articles not yet included in the search strategy. Two authors (DMB and YTK) conducted a full-text review, and discrepancies were resolved with a discussion involving a third author (WAB).

### Data extraction

We created an Excel data extraction template with standardised formatting (JBI data Extraction tool). The data extraction template was tested on 50% of the articles and continuously revised as needed. Two investigators (DMB and YTK) independently extracted the data from the included studies. Information gathered included study characteristics (first author's name, publication year, data collection year, study setting, country, design, and limitations), participant characteristics (child age and sex, sample size, comorbidities, cause, neonatal outcomes, frequency of admission, and period of hospitalisation), and outcome of interest.

### Quality assessment

Two authors (DMB and YTK) assessed methodological quality and risk of bias for the included studies. Any discrepancies were resolved through discussion with a third author (WAB). Separate quality assessment JBI tools were employed to evaluate the methodological quality and risk of bias in cohort, cross-sectional, and case-control studies (40). The JBI tool was appropriate to make the risk of bias assessment systematic, reproducible, and transparent. The tool provides specific appraisal checklists for different study designs. Each checklist has clear questions with defined response options, enhancing consistent interpretation among reviewers. The tool is ideal for use by multiple reviewers, supporting inter-rater reliability. These tools included criteria for selecting and comparing study groups, strategies for controlling confounding, the appropriateness of the statistical analysis, follow-up procedures, and the ascertainment of outcomes of interest. Scores on the tools ranged from zero to eleven, with a score of 50% or higher (≥6) on the quality assessment scale considered low risk. Inter-rater reliability between reviewers was substantial, with a Cohen's Kappa of 0.78 [Supplementary file (Supplementary Table 2)].

### Data synthesis and analysis

The extracted data were transferred to Stata 17 software for meta-analysis (41). A meta-analysis was performed for each developmental and educational outcome whenever two or more studies were available. A random-effects model was used to estimate the pooled odds ratios (POR) for developmental and educational outcomes among children whose mothers had postnatal MHrH. To ensure consistency in the meta-analysis, all effect sizes were converted to odds ratios (ORs), which were the most commonly reported effect measures across the included studies. An online effect size converter for meta-analysis was used to perform these transformations (42). The process varied depending on the type of reported effect measure: relative risks were converted to odds ratios using a 2 × 2 contingency table based on available data. The mean differences were first transformed to Cohen's d, which was used, along with its standard error, to calculate the corresponding odds ratio. Correlation coefficients were also transformed to Cohen's d and its standard error, and subsequently into odds ratios. For studies that reported adjusted effect measures, the transformed values were also considered unless otherwise noted. The results were presented using a forest plot, and the effect size was reported with 95% CI (43).

The I-squared (*I^2^*) statistic was computed to evaluate the percentage of total variation across studies (heterogeneity). *I^2^* values of 25%, 50%, and 75% were interpreted as low, moderate, and high heterogeneity, respectively. A *P*-value below 0.05 for the *I^2^* statistic with the Der Simonian and Laird (DL) test indicated substantial heterogeneity (44). Publication bias was assessed by examining the symmetry of the funnel plot (45) and Egger's test with a *p*-value less than 0.05 (46). A sensitivity analysis was conducted to assess the impact of individual studies on the meta-analysis results.

## Results

### Search results

**Figure 1:**
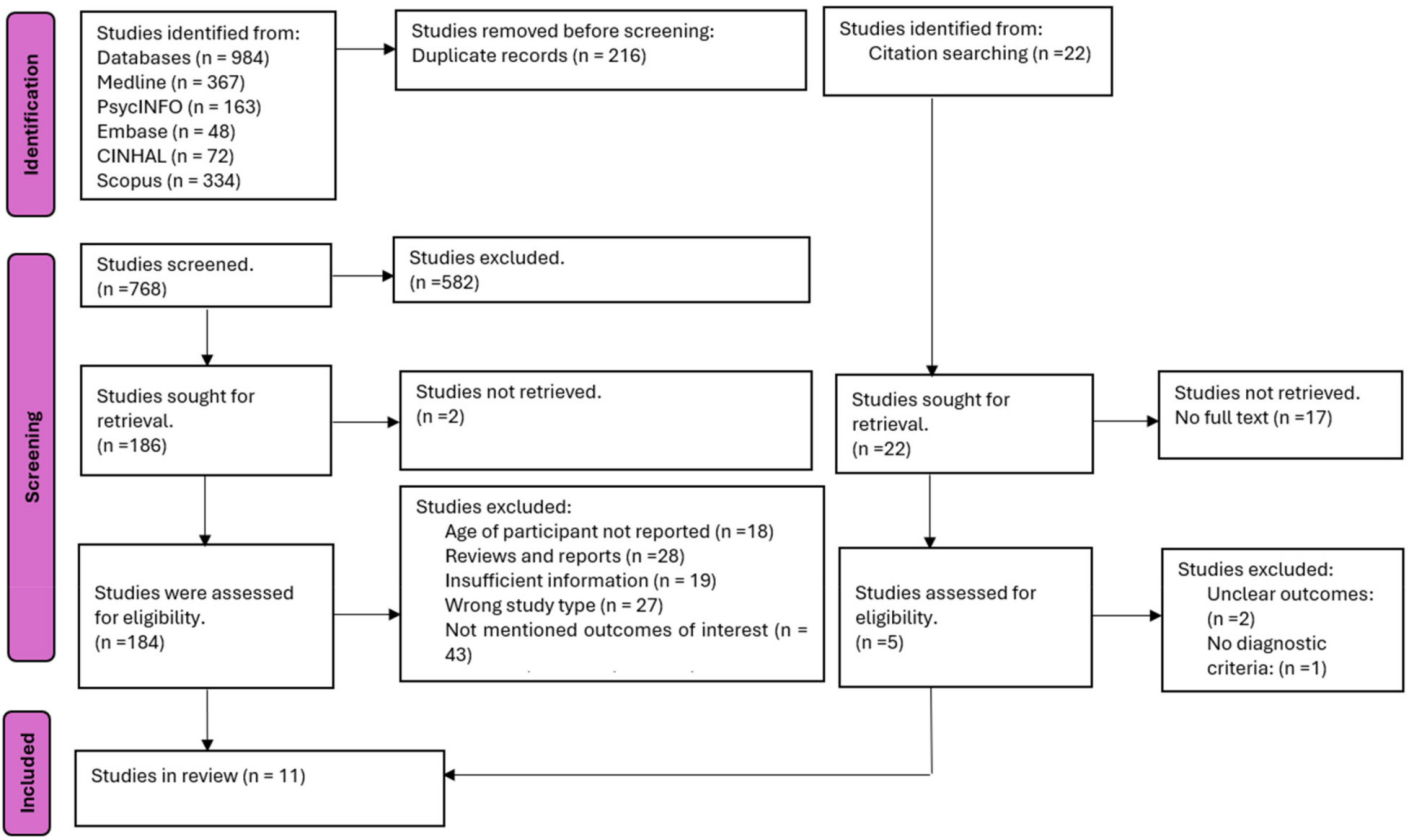
PRISMA flow diagram, 2025.

Our search generated 1,006 records, including 984 records identified from database searches and 22 additional records from citation searches. After removing duplicates and screening, 11 eligible studies (*n* = 535,438) were selected for this review (Figure 1).

### Characteristics of included studies

Eleven primary studies were included in the analysis (35, 36, 47–55). Among these, nine focused on how maternal MHrH within the postnatal period affected childhood development outcomes (35, 36, 47–53). The other two examined the link between postnatal maternal MHrH and childhood education outcomes (54, 55). The studies spanned four continents and were published between 1980 and 2019. Five studies were conducted in Australia (35, 49, 53–55), one in Europe (51), three in the United States of America (USA) (35, 48, 50), and three in Africa (36, 47, 52). Seven studies were retrospective cohort studies (35, 47–49, 53, 54), one was a prospective cohort study (36), one was a case-control study (51), and two were cross-sectional studies (52, 53). The included studies varied in sample size from 83 to 168,528 participants. Three studies reported that participants’ mean ages were 6, 5.6, and 5.5 years (49, 50, 53). Nine of the included studies reported on mental health-related comorbidities and other health conditions in mothers (47–54). Only two studies specifically considered the frequency and severity of these mental health-related conditions (49, 50). Five studies reported neonatal outcomes, including preterm birth, low birth weight, and small for gestational age (36, 47, 48, 50, 52). According to the seven included studies, 50%–60% of the children were male (48–54). Additionally, nine of the included studies performed adjusted analyses (47–54).

Among the nine studies evaluating developmental outcomes, two studies utilised the BSID-III to assess cognitive, language, social, and behavioural/emotional domains of development (36, 47). Another study employed the GMA to assess the motor/physical domain of development (52). A separate study utilised the ICD-10 criteria from hospital inpatient records to assess social, communication, and behavioural domains of development (51). The remaining five studies employed the EDI/AEDC to assess physical, cognitive, social, emotional, and communication domains of development (35, 48–50, 53). Among studies assessing childhood educational outcomes, two Australian studies utilised results from National Assessment Program – Literacy and Numeracy (NAPLAN) tests administered to all Australian students in Years 3, 5, 7, and 9 of schooling. Standardised scores from these tests are used to identify below-standard academic performance (i.e., not meeting National Minimum Standards (NMS)) across measures of numeracy, reading, spelling, and writing (54, 55).

For the measurement of MHrH, two studies used the Diagnostic and Statistical Manual of Mental Disorders, Fifth Edition (DSM-5) among psychiatric inpatients (36, 47). Nine studies employed the International Classification of Diseases, 9th/10th revisions (ICD-9/10), to report diagnoses of mental health and behavioural conditions amongst hospitalised mothers (35, 48–55). Regarding the duration of the postnatal period, seven studies documented maternal MHrH within 12 months of birth (35, 36, 47–49, 52, 53). The remaining four studies specified that maternal MHrH extended up to 18 months after birth (50, 51, 54, 55) (Table 1).

**Table 1:** Characteristics of the study participants .

Author	Country	Sample size	Child characteristics	Admission cause	Comorbidities	Adjusted variables
Burger M, et al.	South Africa	83	1.5 years, preterm (7%)	Anxiety & mood disorders	Mood & psychotic disorders	Not done
Daniels JL et al.	Sweden	31, 920	10 years, male (77%)	Any mental health-related conditions	Schizophrenia, affective, non-affective psychoses, neurotic, personality, & substance misuse.	Child's age, gender, hospital of birth, parents’ age at delivery, socio-economic status, country of birth, & mental health of the father.
Bell MF et al.	Manitoba	68, 274	5.2 years	Any mental health-related conditions	Not reported	Not done
Bell MF et al.	Western Australia	19, 529	5.1 years	Any mental health-related conditions	Not reported	Not done
Burger M et al.	South Africa	112	10–20 wks., preterm (7.2%), small for gestational age (2.8%)	Psychotic disorders	Mood, anxiety, psychotic disorders, substance misuse & HIV	Gestational age
Green MJ et al.	New South Wales	66,045	5 years, male (50.5%)	Any mental health-related conditions	Psychosis, schizoaffective, depression, anxiety, neurotic, personality, substance abuse, other adult onset, other childhood onset, maternal infectious & non-infectious disease.	Sex, English as a second language, socioeconomic status, maternal age, & prenatal smoking
Wall-Wieler E et al.	Manitoba	52, 103	5 years, preterm (7.3%), male (51.2%)	Depression	Not reported	Age at 1st birth, education, social isolation, marital status, smoking & alcohol during pregnancy, income, birth order, child sex, & gestational age
Bell MF et al.	Western Australia	19, 071	5 years, male (50.2%)	Any mental health-related conditions	Mood, anxiety disorder, & substance misuse	Ethnicity, second language status, remoteness, and socio-economic status.
Comaskey B et al.	Manitoba	18,331	5 years, preterm (7.7%), low birth weight (5.2%), male (50.9%)	Depression or anxiety	Depression or anxiety	Genetics, *in utero* influence, attachment, family functioning, income, social resources, low birth weight, neonatal intensive care unit stay, extended birth stay, sex, & maternal age.
Ayano G et al.	New South Wales	168,528	14 years, male (67.36%)	Any mental health-related conditions	Schizophrenia, bipolar, depression, substance misuse, anxiety, chronic hypertension, & diabetes mellitus.	Age, smoking during pregnancy, hypertension, diabetes mellitus, parental education, marital status, occupation, sex, low birth weight, & language spoken at home.
Lin A et al.	Western Australia	91, 522	12 years	Any mental health-related conditions	Schizophrenia, bipolar disorder, major depression, & psychosis	Gender, English second language, socioeconomic status, remoteness, ethnicity, obstetric complications, gestational age, maternal marital status, maternal age, parental age, & paternal mental illness.

### The association between postnatal maternal MHrH and childhood developmental vulnerability

**Figure 2:**
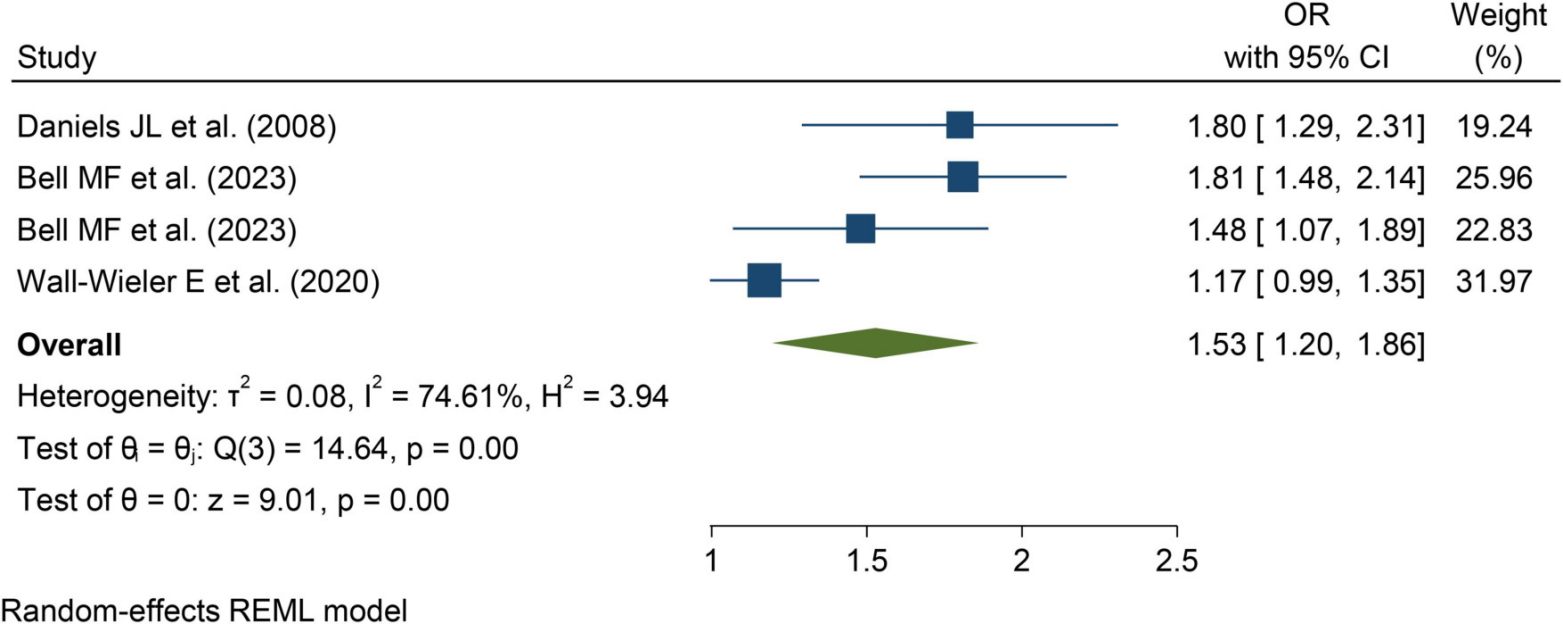
Pooled odds ratios for developmental vulnerability, in any domain, among studies of children whose mothers experience a postnatal hospital admission for a mental health-related condition, 1980-2019 (*n* = 171,826).

The pooled estimate indicated a statistically significant association between postnatal maternal MHrH and developmental vulnerability in any of the five domains (POR = 1.53, 95% CI 1.20–1.86). However, this result should be interpreted with caution due to substantial heterogeneity among the included studies (I^2^ = 74.61%, Cochran's *Q* test (Q (3) = 14.64, *p* < 0.001), and *τ*^2^ = 0.08) (Figure 2).

**Figure 3:**
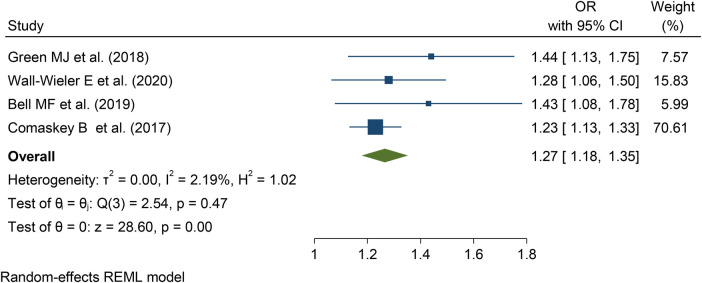
Pooled odds ratios for developmental vulnerability in the social domain among studies of children whose mothers experience a postnatal hospital admission for a mental health-related condition, 1980-2019 (*n* = 171,826).

The pooled estimate indicated a statistically significant association between postnatal maternal MHrH and developmental vulnerability in the social domain (POR = 1.27, 95% CI: 1.18–1.35). There was low and non-significant heterogeneity among the included studies (I^2^ = 2.19%, Cochran's *Q* test (Q (3) = 2.54, *p* > 0.05), and *τ*^2^ = 0.00) (Figure 3).

**Figure 4:**
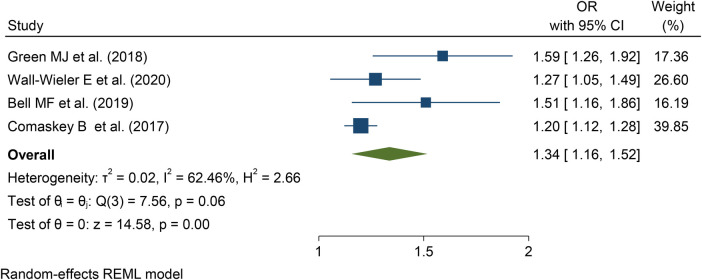
Pooled odds ratios for developmental vulnerability in the emotional domain among studies of children whose mothers experience a postnatal hospital admission for a mental health-related condition, 1980-2019 (*n* = 155,550).

The pooled estimate indicated a statistically significant association between postnatal maternal MHrH and developmental vulnerability in the emotional domain (POR = 1.34, 95% CI: 1.16–1.52). Although the Q tests show non-significant heterogeneity, the I^2^ statistic indicated substantial heterogeneity (I^2^ = 62,46%, Cochran's *Q* test (Q (3) = 7.56, *p* > 0.05), and *τ*^2^ = 0.02). Given the limited number of studies included, the Q test may lack power to detect true heterogeneity, and the results should be interpreted cautiously (Figure 4).

**Figure 5:**
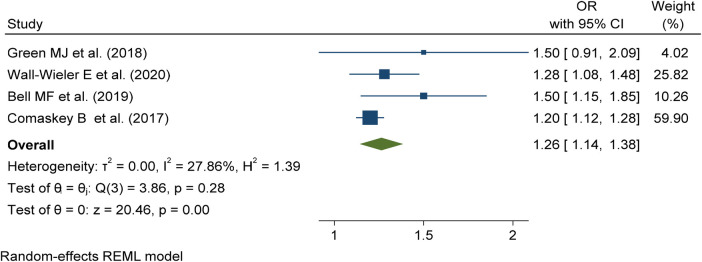
Pooled odds ratios for developmental vulnerability in the physical domain among studies of children whose mothers experience a postnatal hospital admission for a mental health-related condition, 1980-2019 (*n* = 155,550).

The pooled estimate indicated a statistically significant association between postnatal maternal MHrH and developmental vulnerability in the physical domain (POR = 1.26, 95% CI: 1.14–1.38). There was low and non-significant heterogeneity among the included studies (I^2^ = 27.86%, Cochran's *Q* test (Q (3) = 3.86, *p* > 0.05), and *τ*^2^ = 0.00) (Figure 5).

The bias-adjusted effect estimate indicated that the odds of vulnerability in the social and emotional domains of development were 1.21 times (POR = 1.21, 95% CI:1.16, 1.31, *p* < 0.001) and 1.22 times (POR = 1.22, 95% CI: 1.01–1.44), *p* < 0.01) higher, respectively, among children whose mothers experienced postnatal MHrH compared to those whose mothers did not. However, there was no statistically significant association between postnatal maternal MHrH and vulnerability in the cognitive (POR = 2.41, 95% CI: 1.00–3.82, I^2^ = 99.15%, Cochran's *Q* test (Q (5) = 294.44, *p* < 0.01, and *τ*^2^ = 3.04)) and communication (POR = 1.21, 95% CI: 0.96–1.47, I^2^ = 70.88%, Cochran's *Q* test (Q (5) = 11.14, *p* < 0.05, and *τ*^2^ = 0.05)) domains of development. A higher albeit statistically uncertain odds of developmental vulnerability in the cognitive domain was found to be associated with postnatal MHrH (POR = 2.41, 95% CI: 1.00–3.82, I^2^ = 99.15%, Cochran's *Q* test (Q (5) = 294.44, *p* < 0.001, and *τ*^2^ = 3.04)). These results should be interpreted cautiously due to substantial heterogeneity among the included studies [Supplementary file 1 (Supplementary Figures 2–3)].

### Publication bias

The funnel plot's asymmetric distribution suggests publication bias in the included primary studies [Supplementary file 1 (Supplementary Figures 4–9)]. Additionally, Egger's test (*p* < 0.05) indicates significant study-level bias when assessing developmental vulnerability in children whose mothers experienced MHrH within the postnatal period [Supplementary File 1 (Supplementary Table 3)]. However, Egger's test revealed no considerable evidence of publication bias for vulnerability in any, social or physical development domain.

### Trim and fill analysis

A trim-and-fill analysis was conducted to estimate the bias-adjusted effect. The asymmetry observed in the funnel plots for vulnerability in the social and emotional development domains was corrected after accounting for the theoretical inclusion of two missing studies using the trim-and-fill method.

### Sensitivity analysis

A leave-one-out sensitivity analysis was conducted across the developmental domains to evaluate the influence of individual studies on pooled effect estimates and heterogeneity. Results varied by domains, with specific studies contributing disproportionately to the effect size and between-study variability. Across domains, studies with higher weight or unique characteristics (e.g., specific outcomes and study design) notably influenced pooled estimates and heterogeneity. In particular, *Comaskey B* et al.*, Wall-Wieler* et al.*, Bell MF* et al.*, and Green MJ et al*. consistently contributed to between-study variability [Supplementary file 1 (Supplementary Figures 10–15)].

### The association between postnatal maternal MHrH and below-average academic performance

There was a statistically significant association between postnatal maternal MHrH and below-average academic performance in any (POR = 1.57, 95% CI: 1.07–2.07, *I^2^* = 84.62%, Cochran's *Q* test (Q (1) = 6.50, *p* < 0.05, and *τ*^2^ = 0.11)), spelling (POR = 1.48, 95% CI: 1.01–1.95, *I^2^* = 76.69%, Cochran's *Q* test (Q (1) = 4.92, *p* < 0.05, and *τ*^2^ = 0.09)), and writing (POR = 1.37, 95% CI: 1.0–1.70, *I^2^* = 60.38%, Cochran's *Q* test (Q (1) = 2.52, *p* > 0.05, and *τ*^2^ = 0.03)) measures. However, there was no statistically significant association between postnatal maternal MHrH and below-average academic performance in numeracy (POR = 2.02, 95% CI: 0.37–3.68, *I^2^* = 97.64%, Cochran's *Q* test (Q (1) = 42.44, *p* < 0.01, and *τ*^2^ = 1.39)), and reading (POR = 1.57, 95% CI: 0.59–2.55, *I^2^* = 94.95%, Cochran's *Q* test (Q (1) = 19.80, *p* < 0.01, and *τ*^2^ = 0.47)) measures. These results should be interpreted cautiously due to substantial heterogeneity among the included studies. Given the observed heterogeneity, the small number of included studies limited our ability to employ meta-regression, subgroup analysis, and sensitivity analysis [Supplementary file 2 (Supplementary Figures 16–S20)].

### Publication bias

The asymmetric distribution in the funnel plot suggests potential publication bias in the primary studies, particularly on reading and numeracy education measures [Supplemental file 2 (Supplementary Figures 20–24)]. Furthermore, the results of Egger's test (*P* < 0.05) indicate a significant study bias when assessing below-average academic performance in children whose mothers had postnatal MHrH. However, Egger's test revealed no considerable evidence of publication bias for below-average academic performance in any, spelling, or writing domains [Supplementary File 2 (Supplementary Table 4)].

### Trim and fill analysis

A trim-and-fill analysis was conducted to estimate the bias-adjusted effect. The bias-adjusted effect estimates also indicated no significant association between postnatal MHrH and below-average academic performance in numeracy (POR = 1.19, 95% CI: −0.71–3.09, *p* < 0.05) and reading (POR = 1.08, 95% CI: −0.04–2.20, *p* < 0.05).

## Discussion

This is the first systematic review and meta-analysis to synthesise the evidence on the association between postnatal maternal MHrH and the risk of adverse childhood development and education outcomes. Our bias-adjusted results revealed that children whose mothers experienced a MHrH within the postnatal period had 53% higher odds of vulnerability in any developmental domain, 21% higher odds of vulnerability in the social, 22% higher odds of vulnerability in the emotional, and 26% higher odds of vulnerability in the physical domains of development compared to children whose mothers did not experience a MHrH in the postnatal period. Likewise, bias-adjusted pooled odds of below-average academic performance in any, spelling, and writing measures were 57%, 48%, and 37% higher among children of mothers with postnatal MHrH compared to those without. However, no statistically significant association was found between postnatal maternal MHrH and vulnerability in the development of communication skills, or with below-average academic performance in numeracy and reading.

Findings from this systematic review reinforce existing evidence-based recommendations (e.g., World Health Organisation) for early screening, comprehensive psychosocial assessment, and the provision of adequate, timely, and family-centred follow-up care and support in community settings to prevent and mitigate the impact of maternal mental health issues on child outcomes. Importantly, effective identification and responses to maternal mental health issues in the perinatal period must go beyond treating the individual mother alone. Screening before, during, and after pregnancy, combined with a whole family approach that addresses both parental mental health issues and improves mother-child interaction, is essential (56–58). However, some women may be reluctant to disclose their parenting or family circumstances due to stigma or fear of child protection involvement (59). Therefore, effective support requires a sensitive, respectful, and holistic approach that acknowledges the complexities of parental mental health conditions and priorities the needs of both mothers, children, and their families (60).

Where hospitalisation is considered necessary, it is important that mothers have access to holistic, multidisciplinary care through specialist mother-baby units, where co-admission of children is supported (61). This ensures that mothers have access to specialist inpatient care and treatment, whilst remaining with and being supported to continue providing care for their child. Mother-baby units deliver comprehensive, whole-family-centred care aimed at strengthening mother-infant relationships and supporting parenting capacity. Within the unit, ongoing physical, developmental, and socio-emotional assessments of the infant facilitate early identification of developmental and related problems, enabling timely interventions (62). However, access to such services remains limited, particularly due to the scarcity of publicly funded inpatient mother-baby units (61).

While this systematic review has integrated the latest evidence on the association between maternal postnatal MHrH and child development and educational outcomes, it is crucial to consider certain limitations. In this review, all included studies were evaluated using the JBI quality assessment tool and found to be of high quality, with scores ranging from 55% to 80%. However, while each study received a high-quality designation, there were variations in quality scores across critical areas, including the selection and comparability of study groups, strategies for controlling confounding factors, the appropriateness of statistical analysis, follow-up procedures, and the ascertainment of outcomes. The review minimizes the risk of systematic bias by including studies from various settings and employing different methodologies. This diversity enhances the generalisability of findings and reduces the likelihood that context-specific or method-specific biases drive results. For example, consistent results across varied designs and populations increase confidence in the robustness of the association. However, such diversity also introduces heterogeneity, which recommends cautious interpretation of the findings. It is worth remembering that publication bias could explain the observed heterogeneity. Despite the large cumulative sample size, the small number of included studies limited the statistical power to detect publication bias and heterogeneity (63). Consequently, further analysis to investigate heterogeneity, such as meta-regression and subgroup analysis, could not be performed (64). The observed heterogeneity may arise from several sources that can influence the pooled estimates (65). First, developmental outcomes were assessed using different tools, each measuring different developmental domains at different ages. Because these tools capture distinct aspects of child development, the magnitude of associations may vary across studies, increasing variability in effect sizes and leading to wider confidence intervals in the pooled estimate (64). Second, maternal MHrH was identified using multiple diagnostic frameworks, including ICD-9/10 codes, DSM-5 criteria, and hospital medical records. Additionally, studies defined the postnatal exposure period differently, with some using 12 months postpartum and others extending it to 18 months. These differences may lead to inconsistent exposure classification, potentially diluting or inflating the estimated association and contributing to a biased pooled estimate (66). Third, the underlying psychiatric diagnoses varied across studies, such as depression, bipolar disorder, schizophrenia, psychotic disorders, and substance misuse. These conditions differ in clinical severity, symptom profile, and their potential impact on parenting capacity, family functioning, and child outcomes (67). For example, severe psychotic disorders may disrupt parent-child interactions or result in prolonged hospitalisation, potentially leading to stronger effects on child outcomes than episodic mood disorders (68). Pooling these diverse conditions may therefore obscure disorder-specific effects, increase variability between study estimates, and reduce the precision of the pooled estimates (64). Fourth, differences in study designs, including case control, cross-sectional, and retrospective and prospective cohort studies, may affect the ability to control for confounding and measurement bias (69). Finally, variations in population characteristics across continents such as Australia, the United States, Europe, and Africa may also influence the pooled estimates. Differences in socioeconomic conditions, health care systems, and early childhood support services lead to variations in the observed associations across studies (70). Residual confounders may also influence the pooled results. Factors, such as socio-economic status, paternal mental health, family structure, maternal education, social support, child maltreatment, parenting practices, or pre-existing mental health conditions, may influence the relationship between maternal MHrH and child outcomes. Although some studies adjusted for selected covariates, the types and extent of adjustment varied, and some relevant covariates were inconsistently measured or controlled (71). As a result, residual confounding may bias the pooled estimates, potentially leading to either overestimation or underestimation of the true association between maternal MHrH and child outcomes (69).

## Conclusion

This review found that children whose mothers experienced postnatal MHrH were at greater risk of vulnerability in at least one domain of development, as well as below-average performance in at least one area of academic outcomes. Whilst these results should be interpreted cautiously due to substantial heterogeneity among the included studies, there is sufficient evidence to support existing recommendations for early screening, diagnosis, and management of maternal mental health as part of routine postpartum care.

## Data Availability

The original contributions presented in the study are included in the article/Supplementary Material, further inquiries can be directed to the corresponding author.
